# The effectiveness of mental states’ self-regulation of students in the course of educational activities

**DOI:** 10.1192/j.eurpsy.2021.1928

**Published:** 2021-08-13

**Authors:** M. Kartasheva, A. Klimanova, A. Prokhorov, A. Chernov, M. Yusupov

**Affiliations:** General Psychology, Kazan Federal University, Kazan, Russian Federation

**Keywords:** self-regulation, student, educational activity, mental state

## Abstract

**Introduction:**

Studied the psychological mechanisms of mental states’ self-regulation of students in the course of the educational activities: at lectures, seminars and exams.

**Objectives:**

The main aims of the study were: 1. To identify the typical methods and techniques of mental states’ self-regulation and regulatory abilities in everyday and stressful conditions of educational activity; 2. To establish the relationships between the quality of subject training, regulatory abilities and states of students; 3. To study the influence of mental structures (semantic, reflective) on self-regulation and regulatory abilities of students.

**Methods:**

To solve these problems used a bank of 23 techniques, including 303 indicators. 260 1-st year students took part in the research (aged 18-20).

**Results:**

As a result of the research, identified the states typical for lectures, seminars, exams. Comparison of mental states characteristics of humanities students and students of natural sciences did not reveal any differences. Among the mental states of highly effective students, particular importance have the cognitive mental states: interest, thoughtfulness and concentration. Found that the effectiveness of students’ mental states self-regulation affects the productivity of passing the semester exam. The most commonly used methods are introspection (withdrawal), self-control, the use of logic, a positive attitude and search activity. This pattern is typical for both mathematics and psychology students.

**Conclusions:**

Found that students with high self-regulation efficiency more often use a wide range of regulatory technics. Established the properties of personality, providing high efficiency of self-regulation, these are: adequacy, awareness, independence and assertiveness. This work was supported by the RFBR grant № 19-29-07072.

**Disclosure:**

No significant relationships.

